# Huge Intravascular Tumor Extending to the Heart: Leiomyomatosis

**DOI:** 10.1155/2015/658728

**Published:** 2015-05-31

**Authors:** Suat Doganci, Erkan Kaya, Murat Kadan, Kubilay Karabacak, Gökhan Erol, Ufuk Demirkilic

**Affiliations:** Department of Cardiovascular Surgery, Gülhane Military Academy of Medicine, Etlik, 06010 Ankara, Turkey

## Abstract

Intravenous leiomyomatosis (IVL) is a rare neoplasm characterized by histologically benign-looking smooth muscle cell tumor mass, which is growing within the intrauterine and extrauterine venous system. In this report we aimed to present an unusual case of IVL, which is originating from iliac vein and extended throughout to right cardiac chambers. A 49-year-old female patient, who was treated with warfarin sodium due to right iliac vein thrombosis, was admitted to our department with intermittent dyspnea, palpitation, and dizziness. Physical examination was almost normal except bilateral pretibial edema. On magnetic resonance venography, there was an intravenous mass, which is originated from right internal iliac vein and extended into the inferior vena cava. Transthoracic echocardiography and transesophageal echocardiography revealed a huge mass extending from the inferior vena cava through the right atrium, with obvious venous occlusion. Thoracic, abdominal, and pelvic MR showed an intravascular mass, which is concordant with leiomyomatosis. Surgery was performed through median sternotomy. A huge mass with 25-cm length and 186-gr weight was excised through right atrial oblique incision, on beating heart with cardiopulmonary bypass. Histopathologic assessment was compatible with IVL. Exact strategy for the surgical treatment of IVL is still controversial. We used one-stage approach, with complete resection of a huge IVL extending from right atrium to right iliac vein. In such cases, high recurrence rate is a significant problem; therefore it should be kept in mind.

## 1. Introduction

Intravenous leiomyomatosis (IVL) is rare neoplasm characterized by histologically benign-looking smooth muscle cell tumor mass growing within the intrauterine and extrauterine venous system [1]. IVL was first described by Birsh-Hirschfield in 1896 and Durck first presented a case of intracardiac extension of IVL in 1907 [2]. IVL was defined by Norris and Parmley in 1975 in a study of 14 cases [1, 2]. Intracardiac extension occurs in about 10% of cases described and is often clinically undetectable. The majority of the patients present with various nonspecific symptoms such as vaginal bleeding, pelvic pain, dyspnea, syncope, and congestive heart failure. Other rare symptoms include fatigue, abdominal pain, ascites, peripheral edema, and deep vein thrombosis [3]. We describe a case of IVL with right cardiac extension, which was successfully treated with one-stage surgical operation.

## 2. Case Report

**Figure 1:**
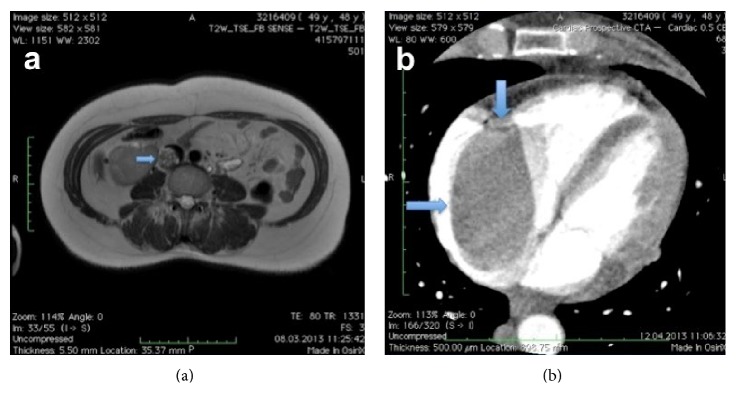
(a) Magnetic resonance venography of the patient at preoperative period (intravenous mass from right internal iliac vein to inferior vena cava, arrowed), (b) view of right atrial mass with venous filling defect (arrowed).

A 49-year-old female patient was admitted to our department with intermittent dyspnea, palpitation, and dizziness. She was diagnosed with uterine leiomyoma four years ago and she had myomectomy operation because she had not wanted total hysterectomy. Nine months ago she was diagnosed with iliac vein thrombosis and receiving already warfarin sodium treatment. Physical examination was almost normal except bilateral pretibial edema. Chest X ray was normal. On magnetic resonance venography (MRV), there was intravenous mass, which was originated from right internal iliac vein and extended into the inferior vena cava (Figure 1(a)). After MRV further examinations such as transthoracic/transesophageal echocardiography (TTE/TEE) and thorax MR were planned.

TTE revealed a mass extending from the inferior vena cava through the right atrium. After TTE we performed TEE and MR. TEE revealed two little membrane-like attachments of the tumor to the right atrial endocardium. Thoracic, abdominal, and pelvic MR showed an intravascular mass, which was concordant with leiomyomatosis. The existing mass was extending from right iliac vein throughout to the right atrium with an obvious venous occlusion (Figure 1(b)). Oral anticoagulation therapy was discontinued, and low molecular weight heparin therapy was started.

**Figure 2:**
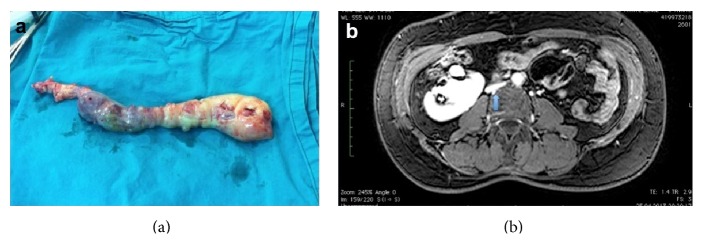
(a) View of the excised mass, (b) magnetic resonance venography of the patient at postoperative period (a relative collapse arrowed, depending on extraction of huge mass).

Surgery was performed through median sternotomy. Beating heart surgery was performed with cardiopulmonary bypass at normothermic range. Cardiopulmonary bypass was established by cannulating the ascending aorta and left femoral vein. Superior vena cava cannulation was performed after pulling out the mass. After right atrial oblique incision, tumor was detached from right atrium. The mass was not attached strongly to the VCI endothelium. During extraction of mass, vena cava superior was clamped. The entire tumor in the IVC and right atrium was extracted from the right atrium (see Supplemental file 1 in the Supplementary Material available online at http://dx.doi.org/10.1155/2015/658728). Operative specimen with intracardiac and intracaval components was 25 cm length, 186 gr weight, and grey-white and rubbery color (Figure 2(a)). Histopathologic assessment was compatible with intravenous leiomyoma.

The postoperative course was uneventful. The patient was discharged on the seventh postoperative day without any problem. Before discharge, we performed cardiac MR and there was no residual tumor. Six months after operation, thoracoabdominal MR was reperformed and there was no residual tumor either. However, there was a relative collapse at VCI, which was depending on the extraction of huge mass from intravascular cavity (Figure 2(b)). The patient's follow-up is still ongoing, and we did not observe any recurrence within the past 14 months.

## 3. Discussion

IVL usually develops in middle-aged women with a history of hysterectomy, invasion in the pelvic vein and extending to the IVC, sometimes to the right atrium. Infiltration of the venous system is an important feature. The mass in the heart and vein structures has no adhesion with the wall of heart and vein [2].

There are two main theories regarding the pathogenesis of IVL. The first one suggests that the neoplasm arises from estrogen-induced smooth muscle cell proliferation in the venous wall of the uterine veins, and the second one suggests that the neoplasm arises from uterine leiomyomas that invade the pelvic veins and inferior vena cava [2, 3]. Our case supports the second theory because the patient had a story of intrauterine leiomyoma.

Clinical signs and symptoms of leiomyoma are related to size and localization of tumor. In our case, symptoms were not acute because of the nature of slow growing tumor. Because of rarity and unusual growth potential of intravenous leiomyoma, several diagnostic tests such as TTE/TEE, computerized tomographic angiography, MRV, and conventional venography should be used to avoid delay in diagnosis and extension. Extension to the right heart chambers can cause valve obstruction, leading to cardiac insufficiency and death [4, 5].

Differential diagnosis of the right atrial mass includes right atrial myxoma, thrombus, and other abdominal tumors with metastasis to the right atrium, such as hepatocellular carcinoma and renal cell carcinoma [4, 5].

Surgical excision is still the best treatment of choice for IVL and complete removal of the tumor is considered essential to prevent a recurrence [4]. Complete resection can be accomplished via a two-stage procedure involving resection of the abdominal/pelvic and intrathoracic components in two separate operations or in a single-stage operation with cardiopulmonary bypass or right heart bypass only [4]. Two-stage operation may have an advantage to avoid the complications of coagulopathy and hemorrhage during cardiopulmonary bypass. However, choosing of surgical procedure is still controversial [4, 5]. Decision should be made with the patient's clinical condition. In our case, we were lucky that we did not need an abdominal approach; all the tumoral mass extracted from right atrium with one-stage operation via sternotomy.

The operative approach requires careful consideration with the aim of complete resection of neoplastic tissue, because recurrence appears to be rare when complete resection is achieved, while incomplete removal leads to high recurrence rates [5]. Recurrence rates are reported as 30% in various studies, with 7-month to 17-year follow-up data [5]. In our case we did not observe any recurrence at 14-month follow-up. Antiestrogenic agents such as tamoxifen may be useful in case of incomplete resection or tumor recurrence, while their efficacy remains still controversial [2, 5].

## 4. Conclusion

Exact strategy for the surgical treatment of IVL is still controversial. However, we used successfully one-stage approach in our patient, with complete resection of a huge IVL extending from right atrium to right iliac vein. In such cases, high recurrence rate is a significant problem; therefore it should be kept in mind.

## Supplementary Material

Tumor was detached from right atrium under cardiopulmonary bypass. The entire tumor in the inferior vena cava and right atrium was extracted from the right atrium. Operative specimen with intracardiac and intra caval components was 25 cm length, 186 gr weight, and grey-white and rubbery color.
